# Inactivating pathogenic bacteria in greywater by biosynthesized Cu/Zn nanoparticles from secondary metabolite of *Aspergillus iizukae*; optimization, mechanism and techno economic analysis

**DOI:** 10.1371/journal.pone.0221522

**Published:** 2019-09-12

**Authors:** Efaq Noman, Adel Al-Gheethi, Balkis A. Talip, Radin Mohamed, Amir Hashim Kassim

**Affiliations:** 1 Department of Applied Microbiology, Faculty of Applied Sciences, Taiz University, Taiz, Yemen; 2 Faculty of Applied Sciences and Technology, Universiti Tun Hussein Onn Malaysia (UTHM), KM1, Jalan Panchor, Pagoh, Muar, Johor, Malaysia; 3 Micro-pollutant Research Centre (MPRC), Department of Water and Environmental Engineering, Faculty of Civil & Environmental Engineering, Universiti Tun Hussein Onn Malaysia, Batu Pahat, Johor, Malaysia; VIT University, INDIA

## Abstract

The inactivation of antibiotic resistant *Escherichia coli* (Gram negative) and *Staphylococcus aureus* (Gram positive) seeded in greywater by bimetallic bio-nanoparticles was optimized by using response surface methodology (RSM). The bimetallic nanoparticles (Cu/Zn NPs) were synthesized in secondary metabolite of a novel fungal strain identified as *Aspergillus iizukae* EAN605 grown in pumpkin medium. Cu/Zn NPs were very effective for inhibiting growth of *E*. *coli* and *S*. *aureus*. The maximum inactivation was optimized with 0.028 mg mL^-1^ of Cu/Zn NPs, at pH 6 and after 60 min, at which the reduction of *E*. *coli* and *S*. *aureus* was 5.6 *vs*. 5.3 and 5.2 *vs*. 5.4 log reduction for actual and predicted values, respectively. The inactivation mechanism was described based on the analysis of untreated and treated bacterial cells by Field emission scanning electron microscopy (FESEM), Energy Dispersive X-Ray Spectroscopy (EDS), Atomic Force Microscopy (AFM) revealed a damage in the cell wall structure due to the effect of Cu/Zn NPs. Moreover, the Raman Spectroscopy showed that the Cu/Zn NPs led to degradation of carbohydrates and amino structures on the bacteria cell wall. The Fourier transform infrared spectroscopy (FTIR) analysis confirmed that the destruction take place in the C-C bond of the functional groups available in the bacterial cell wall. The techno economic analysis revealed that the biosynthesis Cu/Zn NPs is economically feasible. These findings demonstrated that Cu/Zn NPs can effectively inhibit pathogenic bacteria in the greywater.

## Introduction

Greywater represents one of the main alternative sources for fresh water especially in Middle East countries with in arid and semi-arid conditions. However, the impact on health due to the presence of microbial diversity becomes its limitation in utilization of greywater. The microbial load of the greywater has been investigated and reported extensively by many authors in the literature [1]. It has been reported that greywater have *E*. *coli*, Staphylococcus *aureus*, Pseudomonas aeruginosa and Salmonella spp. [1]. The techniques used for the disinfection of the greywater are insufficient for elimination of pathogenic bacteria due to occurrence of resistance among the bacterial species against the chemical and physical disinfection methods [2]. The main problem lies in the ability of pathogenic bacteria to survive during/after the treatment process [1]. Greywater composition is quite different for that of blackwater, the high content of pharmaceutical and personal care products in the greywater might increase the bacterial resistance against disinfection by the chlorination, ozonation and the UV irradiation [1,2]. Moreover, the reverse osmosis filtration with high removal efficiency for the pathogenic bacteria is not suitable for disinfection of greywater due to the presence suspension solids which might block the filter pores. Nanotechnology is a promising method for disinfection of pathogenic bacteria in the wastewater. Among several types of the nanoparticles (NPs) the bimetallic nanoparticles have been given considerable an attention due to their unique properties particularly in designing specific and selective chemical reactions [3–5]. The heterozygosity between two metals increases the stimulation processes in various reactions and thus enhance the efficiency of the product type [6]. Several metals have been used for the synthesis of bimetallic nanoparticles. However, the bimetallic nanoparticles with copper metal have exhibited a highly antimicrobial activity against bacterial cells compared to the silver metal, due to the small size and high surface area which giving them high possibility for interacting with the bacterial cell membranes [7]. Hence, the bimetallic nanoparticles with Cu metals have been used in many applications for the inactivation of pathogenic bacteria in the medical instruments and water treatment [8]. The chemical and physical methods which are the most popular for synthesizing of nanoparticles are associated with environmental pollutions. Therefore, there is a need to develop more eco-friendly technologies with less dangerous and inexpensive compared to the traditional methods [9]. Biosynthesis method has high potential to replace the chemical and physical methods. Many microorganisms, including bacteria, fungi, yeast have the ability to produce nanomaterials [10]. The biosynthesis of NPs has become a new trend in the production of bimetallic nanoparticles. The microorganisms produce metallic nanoparticles outside and inside their cell. The production mechanism is relied on the electrostatic reaction between the positive charge of the metal ions and the negative charge of the functional group available on the surface of the cell wall [11]. Moreover, the peptides in the metabolic products play a critical role in the synthesis of nanoparticles, where the self-assembly of metal ions are occurred due to their bonding with peptides [12].

In fungi, the extra-cellular formation of NPs is taking place through the catalytic effect of the extracellular enzyme and metabolite products which have several reducing agents such as naphthoquinones and anthraquinones [13]. The synthesis of NPs by fungi is more beneficial compared to other microorganisms. This is because fungi have simple requirement for their growth and produce significant number of extracellular enzymes compared to bacteria which accelerate the reduction of metal salts to metal NPs [14]. *Aspergillus flavus*, *A*. *japonicas*, *Cylindrocladium floridanum*, *Penicillium oxalicum*, *Phanerochaete chrysosporium*, *Rhizopus oryzae*, and *Trichoderma reesei* are the most common fungi which are used for synthesizing NPs [15–20]. However, the biosynthesis of bimetallic Cu/Zn NPs from fungi have not been reported before, while the optimization of Cu/Zn NPs in inactivating pathogenic bacteria has not yet being investigate. However, a study conducted by Mohammadi-Aloucheh et al. [21] had observed the effect of the cell surface against antimicrobial activity using Cu/Zn NPs that biosynthesized from *Vaccinium arctostaphylos*. Cu/Zn. The optimization of bacterial inactivation by NPs might enhance the antimicrobial activity of NPs against pathogenic bacteria. In addition, an understanding of the inactivation mechanism and the techno economic analysis using NPs might contribute effectively in making this method more applicable in the water and wastewater treatment plants. This study aims to optimize the inactivation of *E*. *coli* and *S*. *aureus* seeded in greywater by Cu/Zn NPs using response surface methodology (RSM). The inactivation mechanism was investigated, while the economic feasibility for the application of Cu/Zn NPs in a large scale was evaluated based on the techno economic analysis in comparison to the traditional methods.

## Materials and methods

### Isolation of *Aspergillus iizukae* EAN605 strain

*Aspergillus iizukae* EAN605 was isolated from the peat soil sample by culture-based method using standard serial dilution spread plate method on Potato Dextrose Agar medium for enumeration (PDA, Himedia, India) [22]. The fungal strain was purified using single spore isolation technique [23]. The fungal isolate was identified based on the 28 LrDNA. The amplification of DNA was performed using PCR thermal cycler "Veriti 96 Well Thermal Cycler" (Applied Biosystems, USA). The positive and negative sense of primers were amplified the targeted of D1/D2 sequence region (forward primer (F63) 5`GCATATCAATAAGCGGAGGAAAAG3 and reverse primer (LR3) 5`GGTCCGTGTTTCAAGACGG 3`) as described by a study performed by Fell et al. [24]. The sequences were aligned and analysed using NCBI BLAST to identify the species based (http://www.ncbi.nlm.nih.gov/blast) and deposited in gene bank with accession No. MK517570 (https://www.ncbi.nlm.nih.gov/nuccore/MK517570).

### Bacterial strains and antibiotic resistance

*E*. *coli* and *S*. *aureus* were isolated from greywater samples on selective media based on the method described in the previous work [1,25]. The bacterial isolates were purified as accordance to APHA 9225B [22] (S1 Fig). The identification of the bacterial strains were performed based on the morphological, cultural characteristics and biochemical tests (API 20 Staph (Ref. 20 500), API 20NE (Ref 07224 B) and RapiD 20E (Ref 12134 A) (BioMerieux, SA-France) as described by the manufacturers.

### Preparation of fungal supernatant

A pure culture of *A*. *iizukae* EAN605 was sub-cultured in a sterilized basic mineral medium (Hi Media, India) supplement with 10 g of pumpkin peels as a sole carbon source. The inoculated medium with the fungal strain was incubated at room temperature (25±2°C) for 10 days. The fungal mycelium growth was separated by centrifugation at 13,000 rpm for 10 min at 4°C. The supernatant was collected and kept at 4°C until further uses.

### Biosynthesis and characteristics of Cu/Zn nanoparticles

The Cu/Zn NPs were selected to be used in this work after a primary screening for different NPs synthesized with Ag, Cu, Zn, Mn and their combinations (Ag, Cu, Zn, Mn, Ag/Cu, Ag/Mn, Ag/Zn, Cu/Zn, Cu/Mn, Zn/Mn) to choose the most effective NPs which have high antimicrobial activity (Table 1). The Cu/Zn bimetallic NPs were synthesized in 200 mL of distilled water according to the principle described by Ghorbani and Rashidi [26] with modifications. A fixed volume (100 mL) of each metal ions solution in 250 mL of conical flask was mixed, and then 4 mL of the fungal supernatant was added. The flask was left at room temperature (25±2°C) for 24 hrs. In order to check the formation of Cu/Zn NPs, 5 mL of the Cu/Zn solution before and after the mixing with the fungal strain supernatant as well as after 24 hrs post-mixing was scanned for the absorbance using UV-Vis spectrophotometer (DR6000 Hach, USA) with a wavelength ranging from 190 to 1100 nm.

**Table 1:** Antimicrobial activity of antibiotics and Cu/Zn NPs biosynthesized from the secondary metabolite of a novel fungal strain *Aspergillus iizukae* EAN605 grown in pumpkin medium.

NPs			*E*. *coli* (Inhibition zone, mm and responses)						*S*. *aureus* (Inhibition zone, mm and responses)			
	1 μL	Response	5 μL	Response	10 μL	Response	1 μL	Response	5 μL	Response	10 μL	Response
**Ag**	7	R	7	R	7	R	8	M	10	M	11	M
**Cu**	7	R	11	M	11	M	13	S	15	M	15	S
**Mn**	7	R	7	R	7	R	7	R	7	R	7	R
**Zn**	7	R	7	R	7	R	7	R	7	R	7	R
**Ag/Cu**	15	S	20	S	20	S	12	M	12	M	15	S
**Ag/Mn**	7	R	10	S	10	M	9	M	10	R	12	M
**Ag/Zn**	10	M	15	S	18	S	10	M	12	M	20	S
**Cu/Mn**	7	R	7	R	7	R	7	R	9	M	10	M
**Cu/Zn**	10	M	19	S	22	S	10	M	15	S	20	S
**Mn/Zn**	13	S	15	S	20	S	16	S	18	S	18	S
Cu/Zn*	30	S	30	S	30	S	30	S	30	S	30	S
	amoxicillin	ampicillin		ciprofloxacin		tetracycline	erythromycin		Penicillin G		streptomycin	chloramphenicol
***E*. *coli***	S	R		S		R	R		R		M	S
***S*. *aureus***	S	R		S		R	R		R		S	S

*After calcination at 550°C for 3 hrs; R (Resistant); M (moderate); S (Sensitive)

The appearance of peak at the wavelength 274 and 280 nm confirmed the formation of Cu/Zn NPs. The Cu/Zn NPs solution was subjected for the centrifugation at 13000 rpm for 10 min (Rotina 420r Benchtop, Germany). The absorbance of participation was carried re-determined in order to check the presence of Cu/Zn NPs, while the final confirmation of Cu/Zn NPs in the participation was confirmed based on Field Emission Scanning Electron Microscopy with Energy Dispersive X-Ray Spectroscopy, FESEM-EDX (Jeol / Japan- Oxford/ USA).

In order to prepare the powder of Cu/Zn NPs, the previously described procedure was repeated for 10 times with 1 L of NPs solution to get a desirable amount of the Cu/Zn NPs. The participation collected from the centrifugation process was washed with 95% ethanol (Sigma Aldrich, UK), dried at 100°C for 24 hrs and then subjected for calcination at 550°C for 3 hrs [27]. The antimicrobial activity of the dried powder was tested against *E*. *coli* and *S*. *aureus* as detailed in Section 2.5.

In order to study the characteristics of Cu/Zn NPs, one drop from the NPs solution was placed on the upper surface of a small pieces of slide glass (1×1 cm) and left for overnight at room temperature for drying. The slide was used for analysis by FESEM-EDX, Fourier transform infrared spectroscopy (FTIR), atomic force microscopy (AFM) and Raman Spectroscopy (Park System XE-100 / Korea).

### Bioassay of Cu/Zn NPs bioactivity against pathogenic bacteria

The antimicrobial activity of Cu/Zn NPs was determined against *E*. *coli* and *S*. *aureus* grown on Müller-Hinton agar (MHA) using agar diffusion assay. A fixed volume of (0.1 mL, which have 10^6^ cells based on the preparation experiments that were conducted for the inactivation process) *E*. *coli* and *S*. *aureus* suspension (24 hrs cultures) were sub-cultured on the Mueller Hinton Agar (MHA). A sterile cork borer (0.7 cm) was used to bore a well (7 mm in diameter) in each plate. Three concentrations of each NPs (1, 5 and 10 μL) were put into each well in triplicate. The culture agar plates were incubated for 24 hrs at 35°C. The diameters of the inhibition zones around each well contained nanoparticles without bacterial growth were measured. In order to determine the differences between the antimicrobial activities among the different NPs, the size of the inhibition zone was used to classify the susceptibility of *E*. *coli* and *S*. *aureus* towards the NPs. For non-clinical application, the bacterial strains were classified as resistant (7 to 10 mm of inhibition zone), moderately resistant (10 to 12 mm), or susceptible (>12 mm) [28]. For comparison, the susceptibility test by disk diffusion test was conducted according to Al-Gheethi et al. [28] and Morse and Jackson [29]. The positive control used including amoxicillin (10 μg L^-1^), ampicillin (10 μg L^-1^), ciprofloxacin (5 μg L^-1^), tetracycline (30 μg L^-1^), erythromycin (15 μg L^-1^), penicillin (10 μg L^-1^), streptomycin (10 μg L^-1^), and chloramphenicol (30 μg L^-1^). This reference antibiotics type and concentration were selected due to the inhibitory effect against organisms used in this study [30–31]. The Minimum Inhibition Concentrations (MICs) of antibiotics were selected according to the British Society for Antimicrobial Chemotherapy [32].

### Optimization of bacterial inactivation in greywater by Cu/Zn NPs

#### Preparation of bacterial inoculum

Pure culture of *E*. *coli* and *S*. *aureus* were sub-cultured in 1 L of the nutrient broth medium and incubated at 37°C for 24 hrs. Both organisms were selected because they have been considered and used as indicators bacteria in greywater by several authors in the literature [33]. The bacterial cells were separated by centrifugation at 10,000 rpm and 4°C for 10 min. The bacterial cells were washed twice with sterilized physiological saline to remove culture media residues and then suspended in 10 mL of saline water [34].

#### Preparation of greywater samples

The greywater sample (5 L) used in the present study were collected from a village house at Parit Samajan, Johor, Malaysia. The sample was autoclaved at 121˚C for 20 min to inactivate the indigenous microorganism and safe handling. The autoclaved samples were filtrated using membrane filter (Millipore cellulose or Nylon membrane filters; 45 mm diameter, 0.45 mm pore size) according to APHA [22] to remove the suspended solids which might affect negatively on the inactivation process. A fixed volume (500 mL) of autoclaved and filtrated greywater sample was seeded with *E*. *coli* (5 mL, 10^6^ cell mL^-1^), while another sample was seeded with *S*. *aureus* (5 mL, 10^6^ cell mL^-1^). The initial concentrations of *E*. *coli* and *S*. *aureus* in the samples were confirmed based on serial dilution plating method on nutrient agar. Each seeded greywater sample was divided into 20 fractions according to the total numbers of the experiments runs conducted in the optimization process.

### Optimization of bacterial inactivation by Cu/Zn NPs

The inactivation of bacterial cells in the seeded greywater samples by Cu/Zn NPs was optimized using the central composite design CCD (response surface morphology) which was selected to create a significant better model compared to other methods of expert design software program. The CCD requires a smaller number of experiments, where 20 experimental runs were performed in the current work to determine the linear and quadratic effect of Zn/Cu NPs concentration (mg mL^-1^) (*x*_1_) (0.01 to 0.1 mg mL^-1^), time (*x*_2_) (10 to 60 min) and pH (*x*_3_) (6 to 8) as well as the interaction between these factors and their effect on the inactivation of pathogenic bacteria in the greywater samples. The inactivation process using Cu/Zn NPs was performed by adding different concentrations of Cu/Zn NPs for each greywater sample, the pH was adjusted using 0.1 M of NaOH (0.1 M) and 0.1 N of HCl according the RSM suggestions. The inoculated samples were mixed by using magnetic stirrer at 200 rpm. The experiments were conducted in triplicate to obtain the accurate evaluation efficiency of Cu/Zn NPs.

### Enumeration of bacterial cells in the greywater samples

The inactivation of bacterial cells was evaluated based on their ability to grow in NA medium according to APHA [22]. The greywater sample before and after each treatment process was subjected for the serial dilution and then 0.1 ml of each dilution was transferred on the NA medium. The culture plates were incubated at 37°C for 2–3 days to confirm the inactivation of the bacterial cells and the absence of bacterial regrowth [35]. The counts of viable bacterial cells were enumerated in the plates contains 30 to 300 colonies. The numbers of bacterial cells grown were expressed as log_10_ CFU mL^-1^. The log reduction of bacterial cells was calculated according to the Eq (1) as recommended by STAATT [36].

LogReduction(LogKill)=logIT−logRT (1)

where:

**IT** is initial numbers of bacterial cells (log_10_ CFU mL^-1^) in the greywater sample before the inactivation

**RT** is the number of bacterial cells (log_10_ CFU mL^-1^) recovered from the greywater sample after the inactivated by Cu/Zn NPs.

### Mechanism of inactivation process

The bacterial cells morphology before and after the inactivation process was observed using FE-SEM-EDS, FTIT, AFM and Raman Spectroscopy. In brief, 2 mL of bacterial suspension before and after the inactivation process at the optimal conditions (0.028 mg mL^-1^ of Cu/Zn NPs, pH 6, after 60 min) were subjected for the centrifugation at 2000x g for 10 min. The pellets of bacterial cells were subjected for FESEM-EDS (model JEOL JSM-7600F) analysis. The bacterial cells were coated with gold powder with double side carbon tape using auto fine coater (model JEOL JSM-7600F) at 20 mA for 120s and viewed using SEM. The FTIR spectra analysis for each bacterial strain before and after the inactivation process was obtained on Perkin Elmer spectrum 100. The functional groups on the surface of the bacterial cells before and after the production process was determined at the spectral range of 4000 to 600 cm^-1^ at a resolution of 4 cm^-1^, scan number 32 and scan speed of 30 seconds when the pressure gauge is pressed on the sample. The bands were recorded within 30 seconds and used to determine and evaluate the functional groups on the pumpkin peel. The samples for AFM (Park System XE-100/Korea) and Raman Spectroscopy (X-Plora Plus) analysis was prepared by placing the bacterial cells on a surface of a glass slide and then submitted to the AFM analytical laboratory at Minit SCR, UTHM for the analysis.

### Statistical analysis

The experimental runs were conducted in triplicate to ensure the accuracy of the results. Design Expert software was used to analyse the data and investigate the first order response surface equations of the model. The significance of the independent variables on the dependent variables was analysed using ANOVA (*p<0*.*05*). The *R*^*2*^ *adj* was used for checking the fit of the linear model. The interactions between the independent factors and their role in the inactivation of bacterial cells was presented using a three-dimensional graphical representation of the system behaviour, called RSM.

## Results and discussion

### Characterization of biosynthesized Cu/Zn nanoparticles

**Fig 1:**
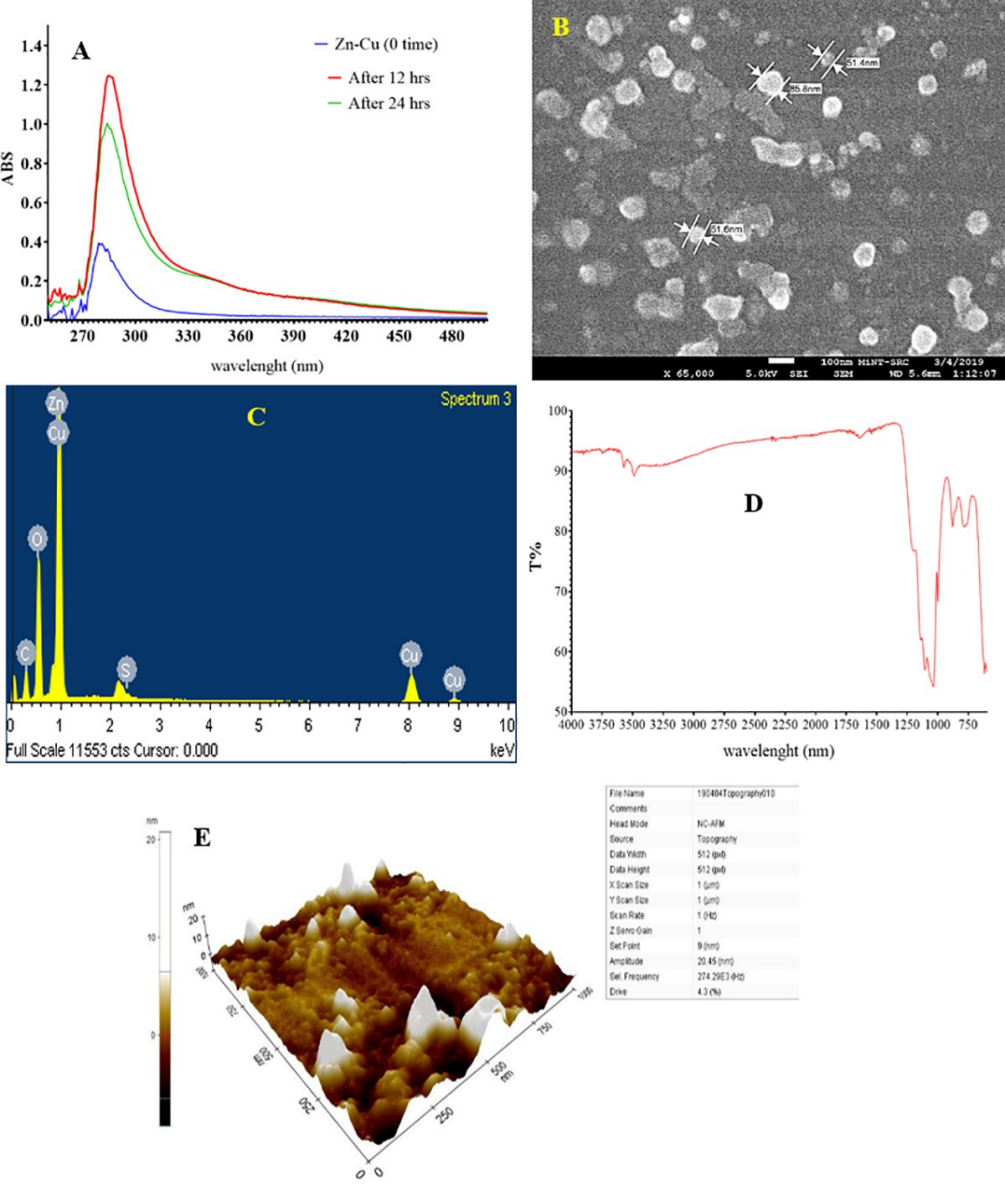
Characteristics of Zn/Cu NPs; a) Absorption spectrum; b) FE-SEM photography; c) EDX analysis of Zn/Cu NPs and Weight % of Zn, Cu elements; d) FTIR spectroscopy of Zn/Cu NPs; e) AFM analysis of Zn/Cu NPs.

A previous work had indicated that the *Aspergillus iizukae* that reported for first time in Malaysia exhibited high efficiency for the decolourization of azo dyes in the wastewater [33]. The results of UV–Vis analysis of Cu/Zn NPs are presented in Fig 1A. The changes in the absorbance of the Cu/Zn solution after 4 mL from the fungal supernatant was added and after the incubation period for 12 and 24 hrs at room temperature (25±2°C) was an indication for forming Cu/Zn NPs. The maximum absorbance at of Cu/Zn NPs after 12 hrs was recorded at 285 nm while was at 284 nm after 24 hrs. The determination of the absorption spectrum is one of the methods used for confirming the formation of NPs [37]. The FESEM analysis revealed that Cu/Zn NPs have a spherical shape with non-uniform distribution and no agglomeration was observed, the size is ranging from 50 to 80 nm (Fig 1B). The EDX detected Cu by 57.73% and Zn by 1.98% of the weight (Fig 1C). The strong intensity and narrow width of Cu/Zn diffraction peaks is an indication for the crystalline shape of the Cu/Zn NPs [38]. The result of FTIR analysis is presented in Fig 1D. It was noted that the peaks are detected at 3571.71, 3488.42, 1646.01, 1105.48 and 1034.74 cm^-1^ which indicate the presence of intramolecular H bonds, C = C, C–O, while peaks below 1000 cm^−1^ are belongs to metal oxides due to inter-nuclear vibrations [39]. The peaks at 3571.71 and 3488.42 might be explains as a result for the presence of OH group [40]. The AFM analysis of Cu/Zn NPs showed that the particles surface has tips and scattered nanoparticles. The 3D image displayed that the nanoparticles have sharp tips of nails on the extract surface (Fig 1E). The antimicrobial activity of Cu/Zn NPs is illustrated in Table 1. The results revealed that among different types of the NPs prepared in the current study, Cu/Zn NPs exhibited high antimicrobial activity, the Cu/Zn NPs occurred more antimicrobial activity after calcination at 550°C (S2 Fig). In comparison to the antimicrobial activity of the antibiotics, it was noted that Cu/Zn NPs exhibited stronger activity against *E*. *coli* and *S*. *aureus*. These results indicated that the NPs has a potential to be used for inactivating pathogenic bacteria in greywater. The concept of non-clinical application or environment used in the present study was used to define the natural antibiotic resistance. The resistant bacteria for one antibiotic might spread their resistance to the other pathogens in the environment. The study of antimicrobial resistance for pathogenic bacteria in the water and wastewater should be conducted based on the concept of non-clinical application [41–42].

### Optimizing the bacterial cells inactivation in greywater by Cu/Zn NPs

The results for the screening of the bacterial inactivation as a response for Cu/Zn NPs (*x*_1_), time (*x*_2_) and pH (*x*_3_) are illustrated in ‎S1 Table. It can be noted that the maximum inactivation (6 log_10_) of *E*. *coli* was recorded with 0.01 mg mL^-1^ of NPs, at pH 8 and after 30 and 60 min. The highest inactivation of *S*. *aureus* (5.21 Log_10_) was noted with 0.055 mg mL^-1^ of NPs, at pH 7 and after 35 min. The different in the responses of *E*. *coli* and *S*. *aureus* for the action of Cu/Zn NPs might be related to the chemical structure of the cell wall. *E*. *coli* is a Gram-negative bacteria with high contents of lipopolysaccharides. *S*. *aureus* is a Gram-positive bacteria with thick cell wall due to the high contents of the saccharides and glutamic acid which might protect the bacterial cells from the negative effects of the NPs [1].

**Fig 2:**
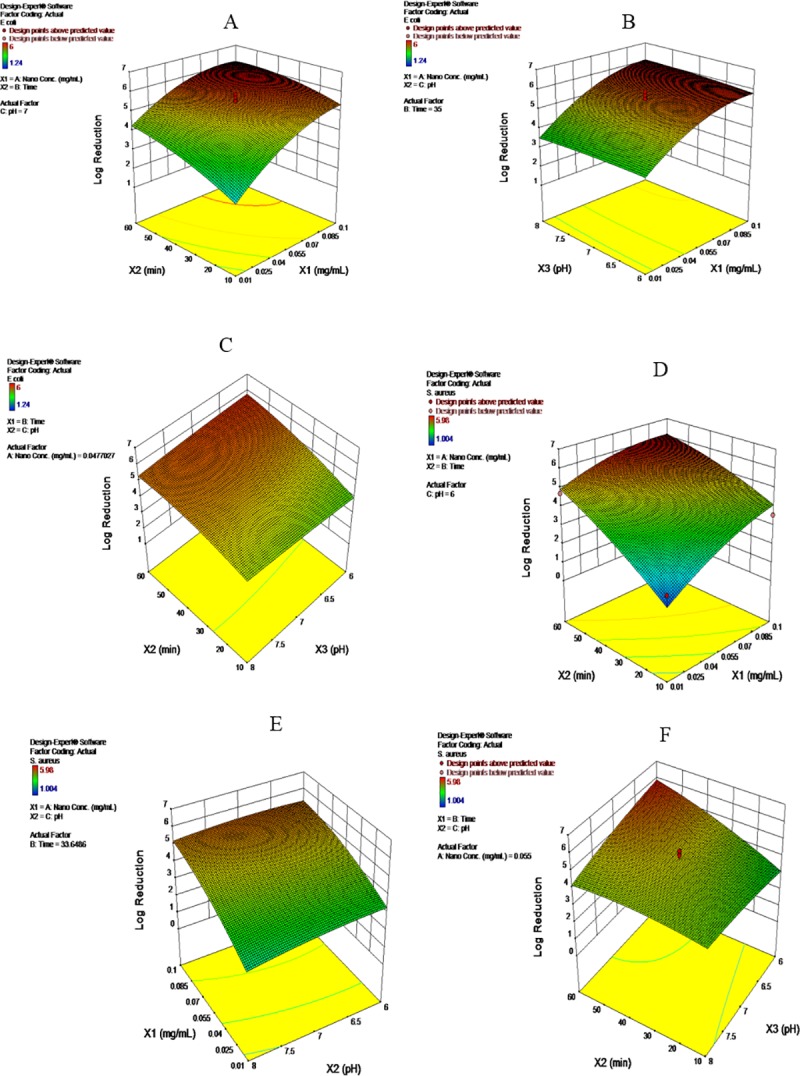
Three-dimensional response surface plot for inactivation of *E*. *coli* (A,B,C) and *S*. *aureus* (E,F,G) in greywater using Zn/Cu NPs as a response of interaction between independent factors, *x*_1_ (Zn/Cu NPs concentration (mg mL^-1^); *x*_2_ (Time, min); *x*_3_ (pH),.

The analysis of the regression coefficient and significance of the quadratic model for inactivating *E*. *coli* and *S*. *aureus* seeded in greywater using bimetallic Cu/Zn NPs showed that both NPs and time was the main independent factors which occurred a significant (P<0.05) inactivation for *E*. *coli* and *S*. *aureus* (S3 Table). NPs and time exhibited a negative significant synergistic interaction (P<0.09) for the inactivation of *E*. *coli*, which does mean that a short time is required for inactivation of *E*. *coli* at high concentrations of NPs. In contrast, the time and pH have a negative significant interaction in their effects on *S*. *aureus* inactivation. Both NPs and time have quadratic contribution in inactivating of *E*. *coli*, but only NPs have the quadratic contribution in inactivating of *S*. *aureus*. These findings indicated that the main effective factors for inactivation of pathogenic bacteria are NPs and time, while pH has a secondary effect. It can also note from Fig 2A that the Cu/Zn NPs has more influence on *E*. *coli* than time. However, the interaction between them at a constant pH 7, increased the inactivation to 6 log_10_ with a short time. NPs and time also exhibited more influence than pH (Fig 2B and 2C). Similar findings were also recorded against *S*. *aureus*, but the maximum reduction in the concentrations of bacteria was 5.9 log_10_ (Fig 2D–2F). In the optimization study of *E*. *coli* inactivation by Asadi and Moeinpour [43], the results also revealed that high inactivation rate of *E*. *coli* within a short time (30 min) was recorded with increasing of silver-coated magnetic nanocomposite concentration from 2 to 10 mg mL^-1^.

Analyses of the variance (ANOVA) of the response surface quadratic model for inactivating *E*. *coli* and *S*. *aureus* in greywater using bimetallic Cu/Zn NPs is presented in Table 2. It was noted that the independent factors contributed by 95.77 and 87.37% with a significant model (<0.05) in the inactivation of *E*. *coli* and *S*. *aureus* respectively.

$$y_{1}=+5.44+1.16\;x_{1}+0.62x_{2}+0.021x_{3}-0.24x_{1}x_{2}+0.11x_{1}x_{3}-0.23x_{2}x_{3}-0.61x_{1}^{2}-0.26x_{2}^{2}-0.056x_{3}^{2}$$ (2)

$$y_{2}=+4.71+1.09\;x_{1}+0.74x_{2}-0.19x_{3}-0.38x_{1}x_{2}+0.12x_{1}x_{3}-0.64x_{2}x_{3}-0.37x_{1}^{2}-0.24x_{2}^{2}-0.14x_{3}^{2}$$ (3)

The best operating parameters for inactivation of both *E*. *coli* and *S*. *aureus* was optimized with 0.028 mg mL^-1^ of Cu/Zn NPs, at pH 6 and after 60 min. The reduction of *E*. *coli* and *S*. *aureus* was 5.6 *vs*. 5.3 and 5.2 *vs*. 5.4 log reduction for actual and predicted values, respectively (S4 Table). In comparison with a previous study conducted by Mohammadi-Aloucheh et al. [21], Cu/Zn nanocomposites with 0.128 mg mL^-1^ revealed high anti-bacterial activities against both *E*. *coli* and *S*. *aureus*. However, this study indicated that a very minimum concentrations of Cu/Zn was required for achieving high inactivation rate. The differences might be related to the nature of inactivation process. Mohammadi-Aloucheh et al. [21] had studied the inactivation in broth medium that contains high nutrients. Instead, this study used the greywater samples for inactivation process.

**Table 2:** Analyses of the variance (ANOVA) of the response surface quadratic model for inactivating *E*. *coli* and *S*. *aureus* in greywater using bimetallic (Cu/Zn) NPs.

Source	Sum of squares		DF	Mean squares		F value		P value	
	*y*_1_*a*	*y*_2_*b*		*y*_1_	*y*_2_	*y*_1_	*y*_2_	*y*_1_	*y*_2_
Model	30.71	31.26	9	3.41	4.37	25.16	7.68	< 0.0001	0.0019
Residual	1.36	4.52	10	0.14	0.45				
Lack-of-fit	*0*.*85*	*3*.*74*	5	*0*.*17*	*0*.*75*	*1*.*68*	4.77	*0*.*2924*	*0*.*0558*
Pure error	*0*.*51*	*0*.*78*	5	*0*.*10*	*0*.*16*				
Total	32.07	35.78	19						

y_1_ (*E*. *coli*); *y*_2_ (*S*.*aureus*); ***a***) R^2^ = 0.9577; Adj R^2^ = 0.9196; ***b***) R^2^ = 0.8737; Adj R^2^ = 0.7600

The presence of nutrient in the broth medium might protect the bacterial cells for the actions of NPs, hence more concentrations are required for achieving high reduction. The inactivation of *E*. *coli* in water by magnetic barium phosphate nanoflakes with embedded iron oxide nanoparticles, achieved 97% within 30 min at 25°C and pH 6 [44]. These findings show that pH 6 is the best for the inactivation of pathogenic bacteria. The high inactivation of bacterial cells at pH 6 might be related to the easy diffusion of NPs through cell membrane and then the high ability to inhibit the metabolic pathway of the bacterial cells. In contrast, at low pH the competition between NPs and proton (H+) is very high. Therefore, the NPs show low affinity to adsorb into the bacterial cell wall and then diffuse into the bacteria cytoplasm [45].

### Inactivation mechanism of bacterial cells by Cu/Zn NPs

**Fig 3:**
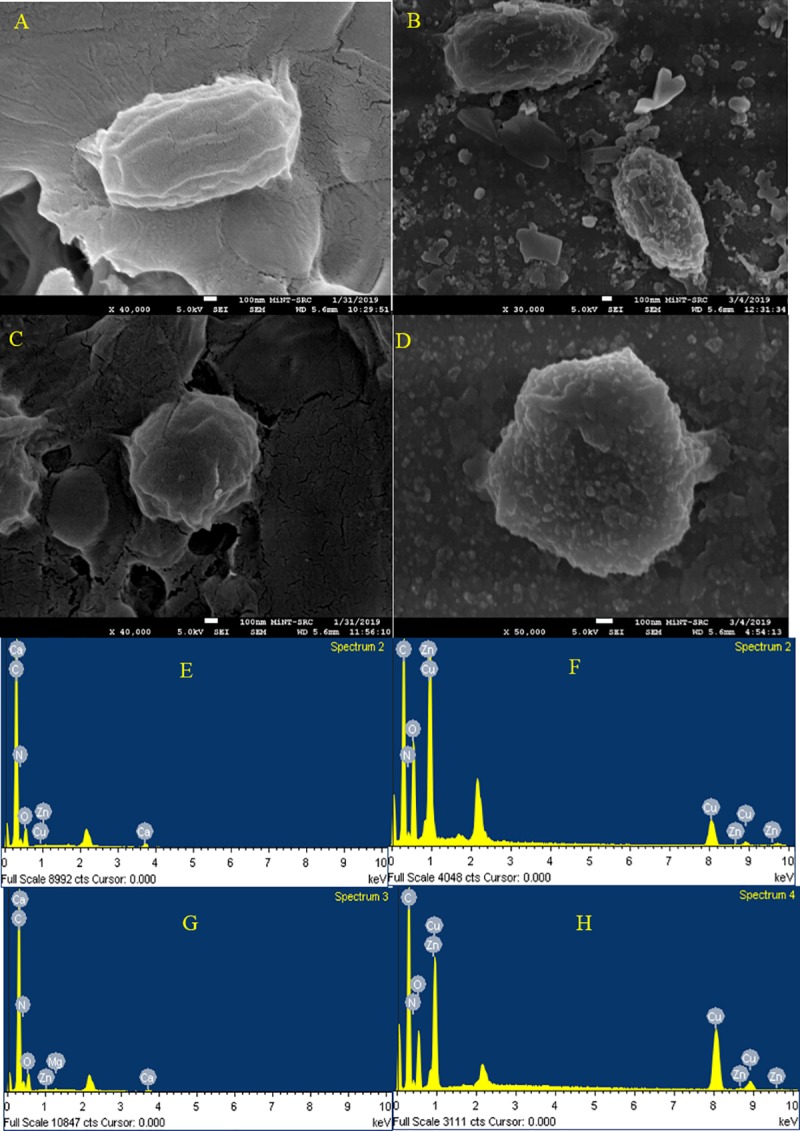
FESEM image and EDX analysis *E*. *coli* and *S*. *aureus* before and after the inactivation process by Zn/Cu NPs, A, E) *E*. *coli* before; B, F) *E*. *coli* after; C, G) *S*. *aureus* before; D,H) *S*. *aureus* after the inactivation process with 0.028 mg mL^-1^ of Cu/Zn NPs, at pH 6 and after 60 min.

**Fig 4:**
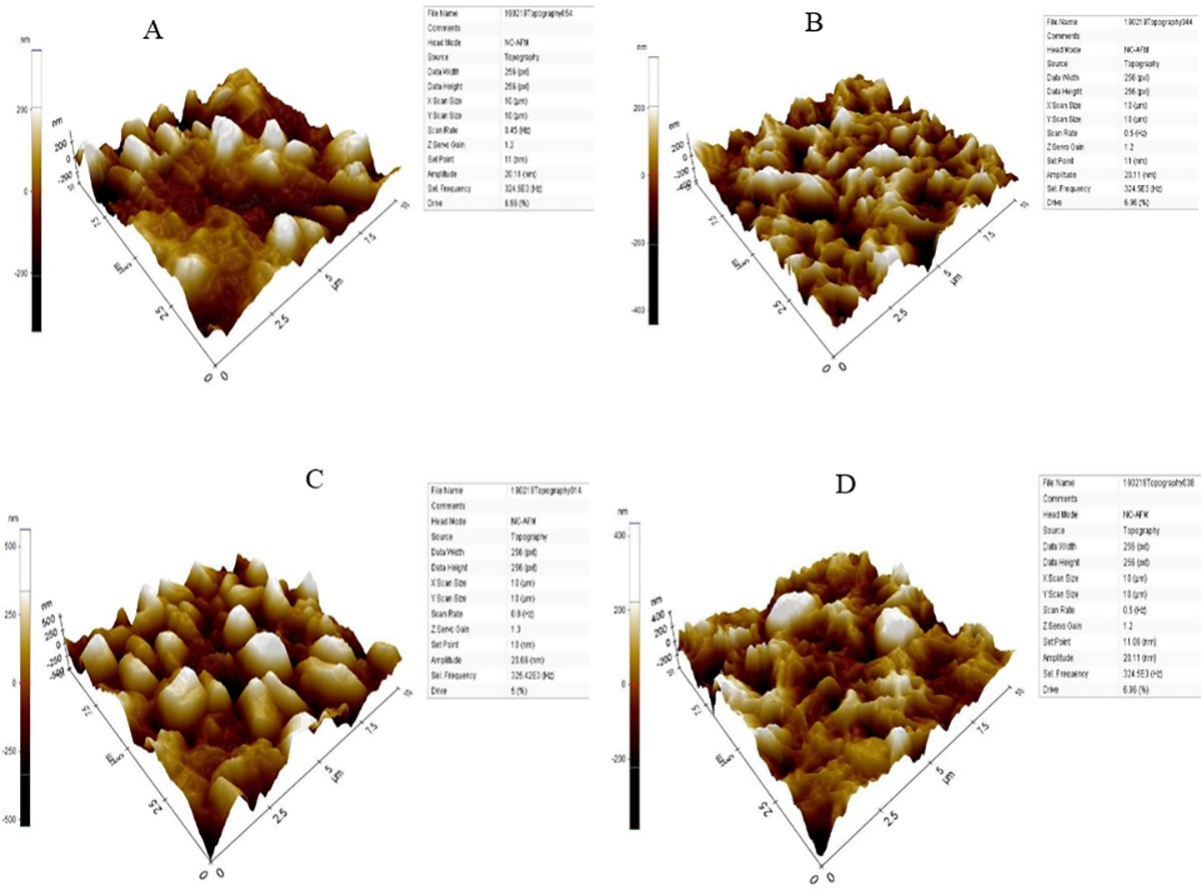
Atomic force micrographs of *E*. *coli* and *S*. *aureus* cell surface before and after inactivation with 0.028 mg mL^-1^ of Cu/Zn NPs, at pH 6 and after 60 min; a) untreated *E*. *coli*; b) treated *E*. *coli*; c) untreated *S*. *aureus*; e) treated *S*. *aureus*.

**Fig 5:**
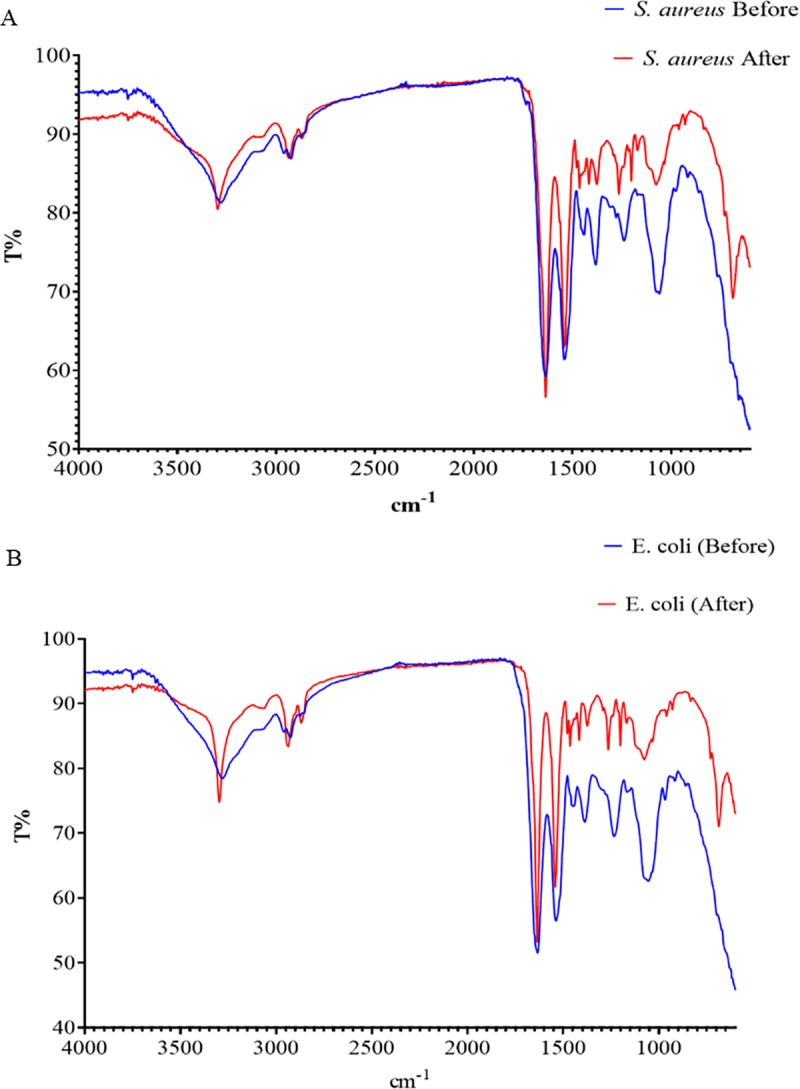
Fourier Transform Infrared Spectroscopy (FTIR) spectrum of *E*. *coli*, and *S*. *aureus* seeded in greywater before after the inactivation process with 0.028 mg mL^-1^ of Cu/Zn NPs, at pH 6 and after 60 min; a) *S*. *aureus*; b) *E*. *coli*.

**Fig 6:**
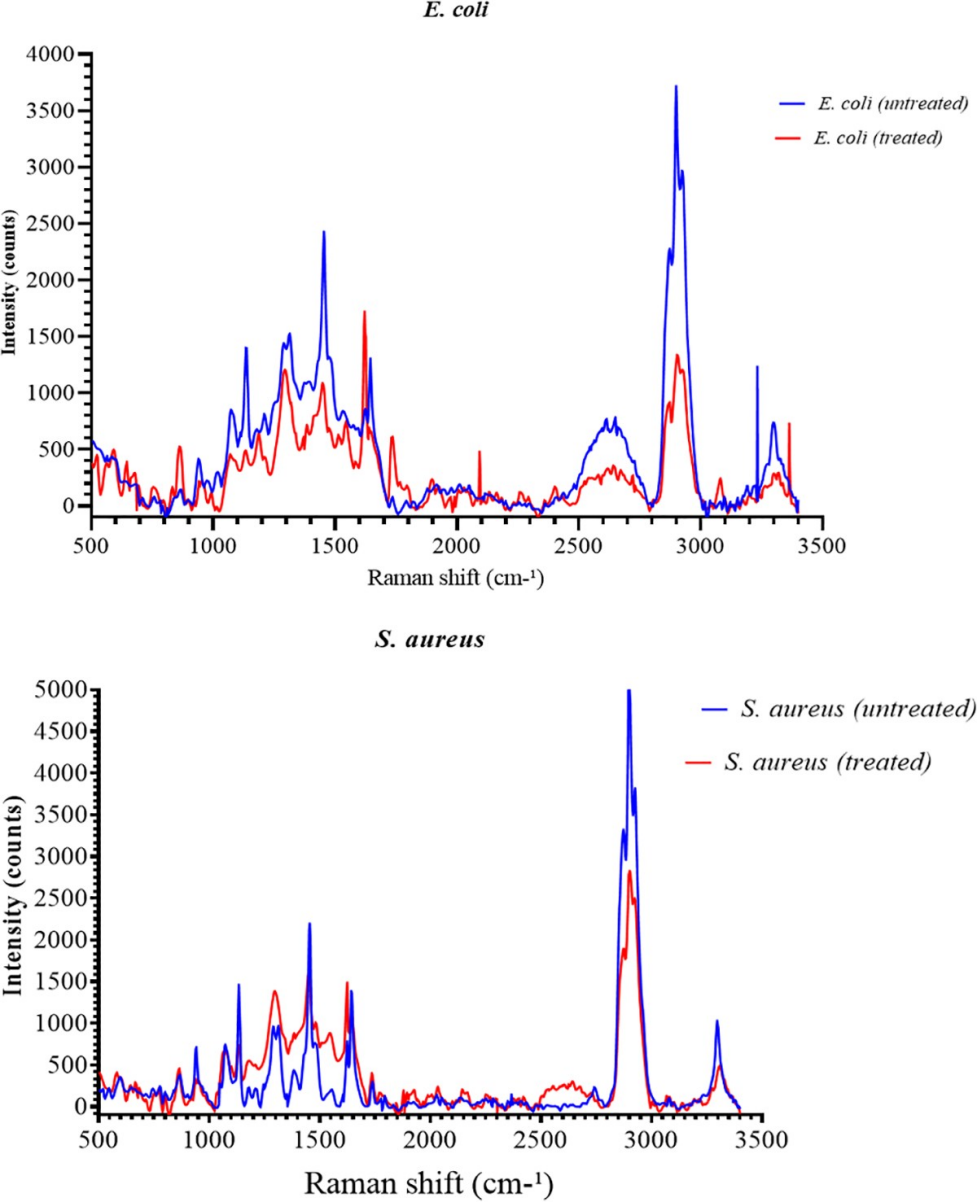
Raman spectroscopy of *E*. *coli* and *S*. *aureus* in greywater before and after inactivation by bimetallic Zn/Cu nanoparticles; a) *E*. *coli*; b) *S*. *aureus*.

The results of FESEM imaging showed that *E*. *coli* seeded in the greywater samples (untreated) has retaining the rod shape with no NPs attached on the surface and without a critical influence on the bacterial cells under the normal environmental conductions (Fig 3A). In contrast, the results in Fig 3B show NPs adhered to the bacterial surface distributed randomly on the bacterial cell wall surface. Similar findings were also noted for *S*. *aureus* (Fig 3C and 3D). These findings are similar as those reported by Mohammadi-Aloucheh et al. [21] which that the Cu/Zn nanocomposite causes obvious cracks in the bacterial membranes. Zhang et al. [46] indicated that the inactivation of bacterial cells by Zn NPs was related to the chemical and physical reactions between Zn and cell membrane components which lead to destruction or blockage the transport channels of the membrane, as well as the direct interaction of Zn NPs with the cell envelope components through electrostatic effect. However, the physical damage caused by the NPs are not detected by FESEM analysis. Therefore, EDS, Raman spectroscopy and FTIR were used to confirm the effect of NPs on the chemical structure of the bacterial cell wall. EDX analysis was used to detect the presence high contents of Zn and Cu as well as Oxygen ions on the surface of *E*. *coli* and *S*. *aureus* after the inactivation process in comparison to the control (Fig 3E–3H). However, the weight of carbon content has reduced, more significant reduction in the nitrogen was observed, where the nitrogen content decreased from 29.28 to 5.95% in the case of *E*. *coli* and from 26.02 to 8.10% in *S*. *aureus*. This reduction indicated that the Cu/Zn NPs might be led to denature the protein contents on the surface of bacteria cell membrane. The negative effects of Cu/Zn NPs on the surface of *E*. *coli* and *S*. *aureus* was confirmed by AFM analysis (Fig 4). The results have revealed the presence of the tips and scattered nanoparticles on the surface of the bacterial cell surface, but it has to mention that AFM was used to confirm the adherence of NPs on the bacterial surface. The AFM shows a rough surface for both bacterial cells after the inactivation process. The FTIR band at 1055.02 and 1058.70 cm^-1^ in the untreated *E*. *coli* and *S*. *aureus* is an indication for C-O-C band that reduced after the inactivation as a result of shifts in the formation of the Zn or Cu+—O- C associates in the electrolytes (Fig 5). The band at 1058 is belongs to the carbohydrate region, while the reduction in this band indicated to the adsorption of Cu/Zn NPs onto the functional group of the carbohydrate compounds [47]. These findings confirm the results obtained from EDX analysis which exhibited a reduction for the carbon content in the treated bacterial cells. The reduction in the function groups of -CH = CH_2_, -CH = CH- and C = CH_2_ in treated bacteria cells as detected between 600 and 1000 cm^-1^, indicates the utilization of the group in the adsorption of Cu/Zn NPs. The advanced analysis of the bacterial cell wall using Raman Spectroscopy confirmed the reduction in the amino groups due to the destruction caused by Cu/Zn NPs (Fig 6). Indeed, the inactivation mechanism of bacterial cells by Cu/Zn NPs based on the chemical analysis of the protein and carbohydrates structure of the bacterial cell wall has not been reported before. Therefore, this study showed the understanding of inactivation mechanism of bacterial cells by using Cu/Zn NPs. Although antibacterial mechanisms have not been fully understood, some studies have reported that the mechanism of oxidative and non-oxidative stress induction as well as metal ion release resembles the antibacterial mechanisms. In addition, the multiple simultaneous mechanisms of NPs action against bacterial cells would require multiple simultaneous gene mutations to develop antibacterial resistance. Therefore, the ability of the bacterial cells to resist NPs is weak [48]. Hence, the discharged greywater after the disinfection process by NPs into the environment have not led to increase the antimicrobial resistance against NPs at least on the short term. To date, no similar finding has been reported.

### Techno-economic analysis for Cu/Zn NPs production and application in inactivating pathogenic bacteria in greywater

**Fig 7:**
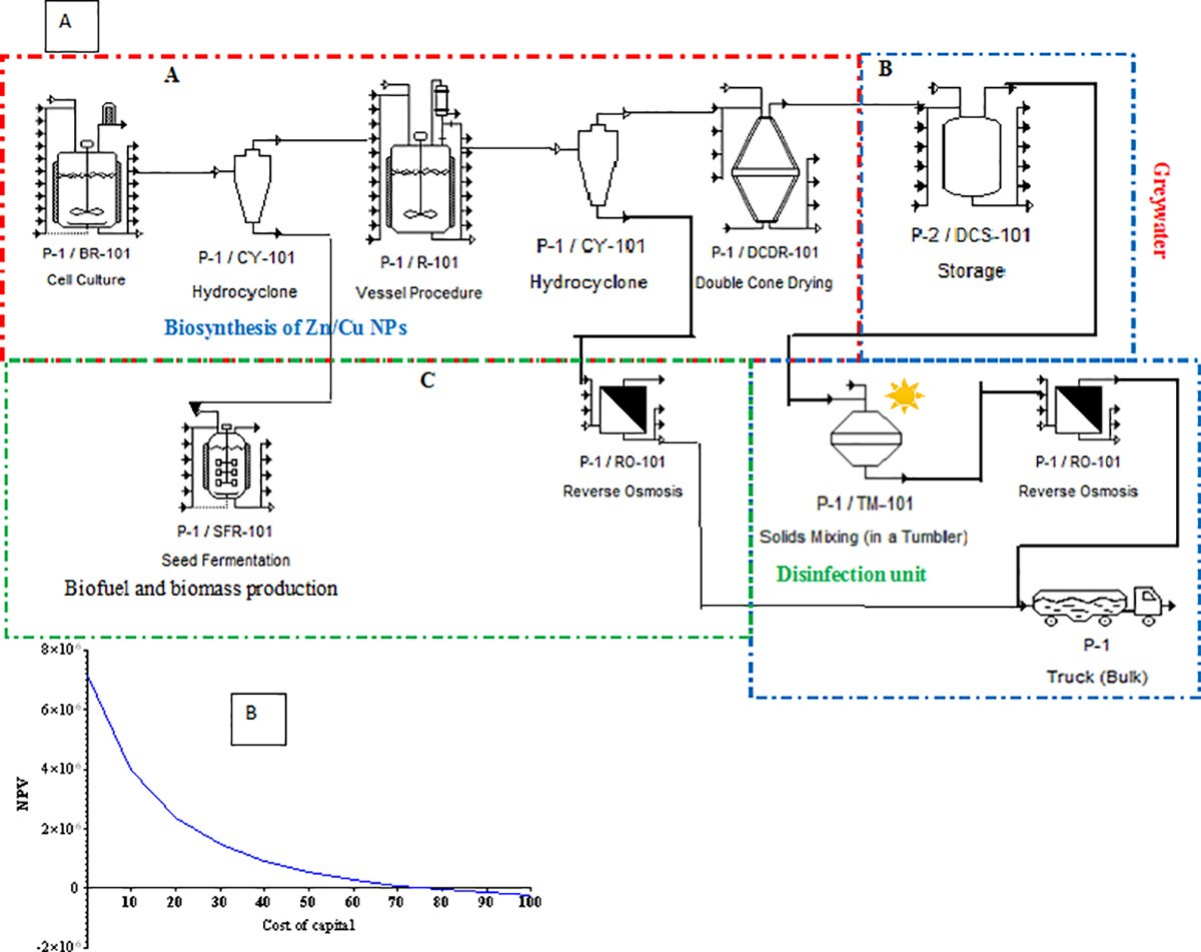
Zn/Cu NPs a) Flowchart of production and application; b) Internal rate of return.

The flow diagram of Cu/Zn NPs production and application was designed using SuperPro Designer and depicted in Fig 7A. The suggested processes consist of three phases which aim to produce, disinfect and dispose and further applications. The disinfected greywater can reused for irrigation. Meanwhile the fungal biomass can be used as a substrate for biofuel generation and bio-sorbents for further environmental application such as heavy metals or azo dye removal by adsorption process. In Phase A, the fungal strain is sub-cultured in a bioreactor with a production medium to generate the secondary metabolite peptides. The fungal mycelium is separated by using centrifugation and the supernatant is used for bio-synthesis of Cu/Zn NPs. The fungal biomass could be used for the fuel production as proposed by Han et al. [49]. In Phase (B), the greywater with bacteria is stored before disinfection process to remove the suspension solid which may effect during the disinfection process by Cu/Zn NPs. The disinfection process should be conducted under the sunlight to enhance the inactivation of pathogenic bacteria by Zn NPs. However, the Cu/Zn NPs have antimicrobial activity in the absence of the sunlight as determined by the agar diffusion method (as mentioned in Section 3.1). The treated greywater should be subjected for reverse osmosis biomass before the final disposal into the environment.

The annual operating time for production and application of Cu/Zn NPs in inactivating pathogenic bacteria in greywater is 7920 h/year (equivalent for 330 operating days). The total capital investment (TCI) for a proposed plant including the fixed capital estimation (FCE) and working capital cost (WCC) (Eq 4) [50–51].

TCI=FCE+WCC (4)

The FCE include equipment purchase cost, process piping, equipment installation, electrical systems, instrumentation and controls, yard improvements, buildings, and construction as well as the working capital cost (WCC) which might represent 6.5% of FCE as suggested by Han et al. [52]. Therefore, the FCE for design a treatment plant with 1000 m^3^/day of capacity reach to USD 950,000.00. (Table 3) The additional costs for the treatment process might reach 50% of the equipment cost which was equivalent to USD 475,000.00 [52]. The TCI of the plant could reach to USD 1,425,000.00. WCC regarded to be 6.5% of the FCE (USD 61,750). Therefore, according to Eq (1), the TCI of USD 1,486,750.00 could be calculated. The techno-economic analysis is one of the best methods for determining the effectiveness of any alternative method for treating the environmental pollution. It has been used for estimating the H_2_ production by fungi from the food wastes and provided very accurate information on the total cost [36,40]. The cost factors associated with the disinfection of greywater in comparison to the physical and chemical treatment process is illustrated in Table 4, which show that the Cu/Zn NPs could be the alternative process for the chemical and physical treatment process.

**Table 3:** Fixed capital estimate (FCE) for biosynthesis of Cu/Zn NPs and application in inactivating pathogenic bacteria in greywater.

Item Code*	Item	Quantity	Percentage of FCE	Cost
P-1/BR-101	Cell culture	1	40	15,000
P-1/CY-101	Centrifuge	2		60,000
P-1/R-101	Vessel procedure	1		30,000
P-1/DCRC-101	Double cone drying	1		15,000
P-2/DCS-101	Storage tank	1		10,000
P-1/TM-101	Solids Mixing	1		80,000
P-1/RO-101	Revise Osmosis	2		20,000
P-1/SRF-101	fuel cell generator	1		50,000
P-1/Track	Track	2		100,000
Total Equipment purchase cost				380,000
Equipment installation			15.79	150,000
Process piping			10.53	100,000
Instrumentation and controls			5.26	50,000
Electrical systems			5.26	50,000
Buildings			12.63	120,000
Yard improvements			2.11	20,000
Construction			8.42	80,000
**TOTAL**				**950,000**

*Refer the item code to Fig 7

**Table 4:** Cost factors associated with the disinfection of greywater by Cu/Zn NPs in a comparison to the physical and chemical treatment process.

Cost factor	Physical	Chemical	Cu/Zn NPs
Heat and energy requirement	2	1	0
Chemicals (acid/alkali/ammonia)	0	2	1
pH neutralization	0	2	0
Detoxification/conditioning	1	2	0
Special reactor construction	2	2	0
**Total**	**5**	**9**	**1**

#### Annual operation cost

The annual operation cost (AOC) for the production and application of Cu/Zn NPs in inactivating pathogenic bacteria include the costs of raw materials (*C*_*RM*_), waste generated from the production process (*C*_*WG*_), utilities (*C*_*U*_) and extra cost (*C*_*E*_) was calculated for annual basis according to Eq (5).

$$AOC=C_{RM}+C_{WG}+C_{U}+C_{E})$$ (5)

The *C*_*RM*_ represent the cost of fungal culture medium which was supplied from the local markets as well as chemicals required for biosynthesis of Cu/Zn NPs, these chemicals were supplied from a local supplier. The *C*_*U*_, which include water and electricity required for the operation process that was estimated based on the price for each unit in the local currency. The *C*_*WG*_ represent the final fungal biomass yield generated from the preparation of fungal supernatant. The solid wastes are considered as a biomass yield with high concentrations of carbohydrates and could be used as bio-sorbents for wastewater treatment as well as for fuel production. In the application of the Cu/Zn NPs for the inactivation of pathogenic bacteria in the greywater, the generated greywater might be used for the irrigation.

Table 5 showed the annual operation cost for the production and application of Cu/Zn NPs. The raw materials cost (*C*_*RM*_) estimated at USD 68,287.20/year. The total cost of utilities is estimated to be USD 4600/year as reported in China [47]. This estimation is similarly reported as in Malaysia. The operating labour requires three operators with an average salary of 10,000 USD/year per operator. The maintenance and insurance were estimated as 2% and 1% of the FCE, respectively according to Vlysidis et al. [53] and Ljunggren and Zacchi [26]. It can be concluded that the annual operation cost for the production and application of Cu/Zn NPs in inactivating pathogenic bacteria is estimated at USD 131,387.20/year.

**Table 5:** Annual operation cost and profitability for biosynthesis of Cu/Zn NPs and application in inactivating pathogenic bacteria in greywater.

	**Component**		**Price**	**Unit**		**Quantity**	**Cost (USD)**
Raw material (chemicals)	Fungal inoculum		37.63	USD /kg		500	10,815.00
	Zinc Acetate Dehydrate		2,59	USD/kg		300	40559.4
	copper Acetate Dehydrate		3.24	USD/kg		100	16912.8
Utilities	Electricity		0.04	USD / kWh		100,000	4000.00
	Water		0.01	USD /m^3^		60,000	600.00
Other costs	Labour		10000	USD / employee		3	30000.00
	Maintenance		2	% of FCE			19000.00
	Insurance		1	% of FCE			9500.00
**Total**							131,387.20
		**Price**			**Quantity**		**Value**
Cu/Zn NPs		USD 569/kg			28 kg		15932
Annual revenue							831650.4
Annual operation cost							131,387.20
local tax							124747.56
Annual profitability							575,515.64

#### Annual profitability and revenue for Cu/Zn NPs synthesis

The revenue for the production of Cu/Zn NPs is to sell the NPs that have several applications in the environmental treatment industries such as disinfection processes of greywater and wastewater. The current price of Cu/Zn NPs (Spherical with 80–100 nm in the diameter size) is USD 569/kg. According to the mathematical model designed by Han et al. [51], the specific cost of Cu/Zn NPs *C*_*Z*_ (kg/USD) is calculated based on the annual costs of capital *C*_*C*_, utilities cost *C*_*U*_, raw material cost *C*_*RM*_ and extra cost *C*_*E*_ divided by the annual Cu/Zn NPs production (Eq 6).

$$C_{Z}=(C_{C}+C_{U}+C_{RM}+C_{E}/E_{P}$$ (6)

This study has shown that the best Cu/Zn NPs dosage for inactivating pathogenic bacteria was 0.028 mg mL^-1^. The total quantity of Cu/Zn NPs required for treating 1000 m^3^ of greywater is 28 kg. No adjustments for pH are required since the optimal condition for the inactivation process was recorded at pH 6, which is closer to the natural greywater. The required quantity of Cu/Zn NPs for treating 1000 m^3^ of greywater is 28 kg. This study also indicates that one L of Cu/Zn solution (contain 0.183 g of Zn C_4_H_6_O _4_ and 0.217 g of C_4_H_10_CuO_6_) with 20 mL of fungal supernatant produces 0.5 g of Cu/Zn NPs, therefore, for producing 28 kg of NPs, 60 m^3^ of metal solution with 183.48 kg of Zn C_4_H_6_O_4_ and 217.664 kg of C_4_H_10_CuO_6_) and 1.2 m^3^ of fungal supernatant. The cell culture unit for fungal growth could be designed with 1 m^3^ of the capacity and with 20 kg of pumpkin peel as a carbon and energy source for fungi in the culture medium for each operation in which 1000 m^3^ of greywater will be treated. The revenue for each production process is 28 Kg of Cu/Zn NPs which give USD 15932 of the revenue for each process. The production of fungal supernatant requires at least 5 days for the incubation period. Therefore, the total numbers of the production processes per year is estimated to be 52.2 process which is going to produce 1.4616 ton of Cu/Zn NPs. The annual revenue is estimated at USD 831650.4. The local tax in Malaysia estimated to be (15%) (USD 124747.56) and annual operation cost (USD 131,387.20/year). The annual profitability (after the tax) of Cu/Zn NPs is USD 575,515.64/year (Table 5). Based on the Eq 7, the specific cost of Cu/Zn NPs (spherical shape with 51–100 nm in the diameter size) is estimated to be USD 89.8 per kg which is lower than the market laccase enzyme price USD 569/kg.

In this study, Cu/Zn NPs was economically evaluated based on the net present value (NPV), internal rate of return (IRR) and payback period (PBP) for 10 years of the life time. The internal rate of return (IRR), which indicated the efficiency of the investment, was 80% in this study (Fig 7B). According to the above results and analysis, the Cu/Zn NPs biosynthesis is economically feasible.

## Conclusion

The current work optimized the inactivation of pathogenic bacteria in greywater by using Cu/Zn NPs. The optimal inactivation rate determined at 0.028 mg mL^-1^ of Cu/Zn NPs, at pH 6 and after 60 min, at which the reduction of *E*. *coli* and *S*. *aureus* was 5.6 *vs*. 5.3 and 5.2 *vs*. 5.4 log reduction for actual and predicted values, respectively. The advanced analysis by FESEM, FTIR, AFM and Raman Spectroscopy has confirmed the damaging of the bacterial cells due to the adverse effect of Cu/Zn NPs, which led to the damage of protein and carbohydrate structure of the bacterial cell wall. On the other hand, the techno economic analysis for the biosynthesis of Cu/Zn NPs is economically feasible with 80% of the internal rate of return (IRR). It may provide alternative methods for disinfection of greywater. The effect on the characteristic and efficiency of NPs based on the C, N, P content in pumpkin need to be further investigated.

## Supporting information

S1 Fig Pure culture of *E*. *coli* and *S*. *aureus* on selective culture media; A) *E*. *coli* on MacConkey agar; B) *E*. *coli* on EMB; C) *E*. *coli* on NA; D) *S*. *aureus* on MHA; E) *S*. *aureus* on NA.
(DOCX)

S2 Fig Antibiotics sensitivity test and Antimicrobial activity of Zn/Cu NPs after calcination at 550°C for 3 hrs against pathogenic bacteria in agar diffusion test; E. coli (A1,2) and S. aureus (B1,2).
(DOCX)

S1 Table Coded and un-coded levels of the independent factors investigated in the present work. (DOCX)

‎S2 Table Central composite design arrangement and responses for inactivation of *E*. *coli* and *S*. *aureus* in greywater using bimetallic Zn/Cu NPs. (DOCX)

S3 Table Regression coefficient and their significance of the quadratic model for inactivating *E*. *coli* and *S*. *aureus* seeded in greywater using bimetallic (Zn/Cu) NPs. (DOCX)

‎S4 Table The best operating parameters for inactivation of *E*. *coli* and *S*. *aureus* in greywater using bimetallic Zn/Cu NPs. (DOCX)
