# Supplementary material for: Inactivating pathogenic bacteria in greywater by biosynthesized Cu/Zn nanoparticles from secondary metabolite of Aspergillus iizukae; optimization, mechanism and techno economic analysis
Source: PLoS One. 2019 Sep 12;14(9):e0221522. doi: 10.1371/journal.pone.0221522 (PMC6742378; doi:10.1371/journal.pone.0221522)
Supplement: S1 Fig — Pure culture of E. coli and S. aureus on selective culture media; A) E. coli on MacConkey agar; B) E. coli on EMB; C) E. coli on NA; D) S. aureus on MHA; E) S. aureus on NA. (DOCX) [file pone.0221522.s001.docx]

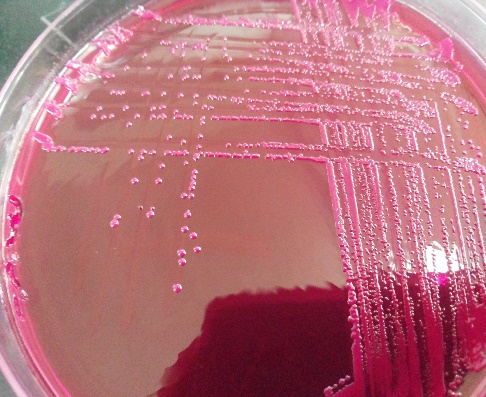

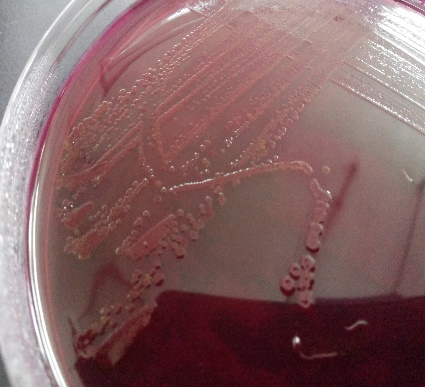

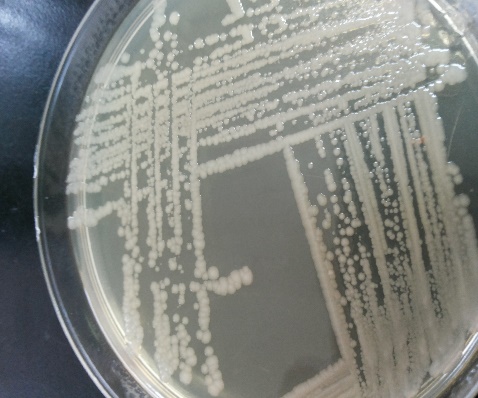


A

B

C


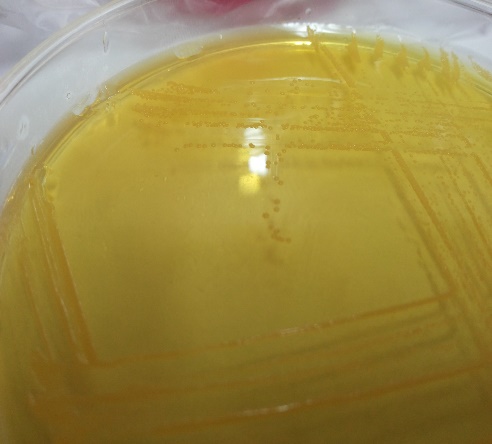

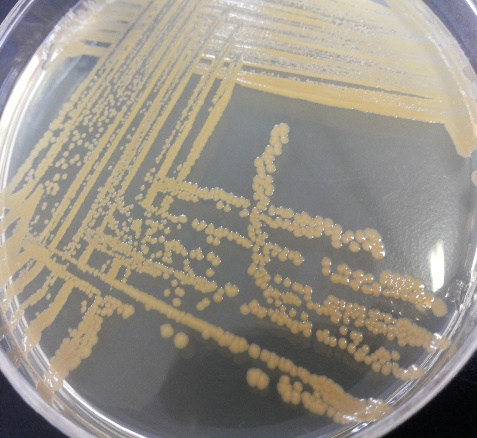


D

E

**S1 Fig.**  Pure culture of *E. coli* and *S. aureus* on selective culture media; A) *E. coli* on MacConkey agar; B) *E. coli* on EMB; C) *E. coli* on NA; D) *S. aureus* on MHA; E) *S. aureus* on NA
