# Supplementary material for: Inactivating pathogenic bacteria in greywater by biosynthesized Cu/Zn nanoparticles from secondary metabolite of Aspergillus iizukae; optimization, mechanism and techno economic analysis
Source: PLoS One. 2019 Sep 12;14(9):e0221522. doi: 10.1371/journal.pone.0221522 (PMC6742378; doi:10.1371/journal.pone.0221522)
Supplement: S2 Fig — Antibiotics sensitivity test and Antimicrobial activity of Zn/Cu NPs after calcination at 550°C for 3 hrs against pathogenic bacteria in agar diffusion test; E. coli (A1,2) and S. aureus (B1,2). (DOCX) [file pone.0221522.s002.docx]

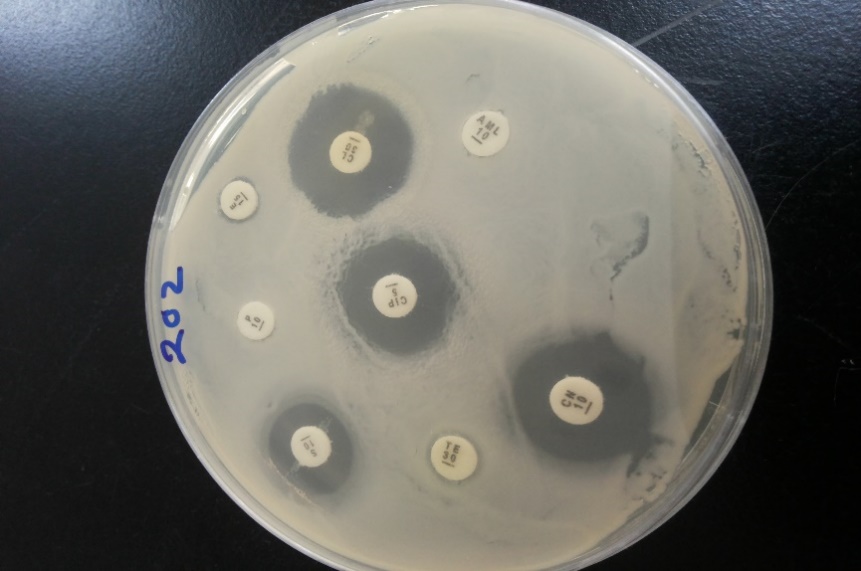

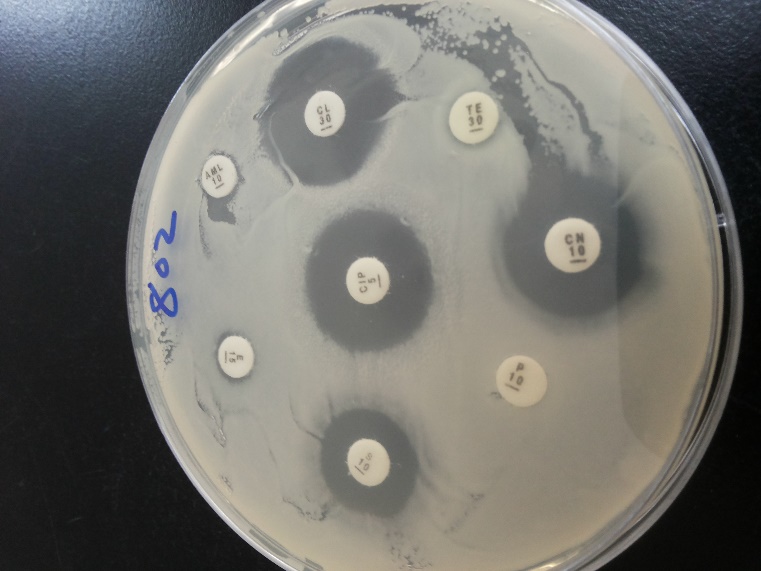


A1

B1


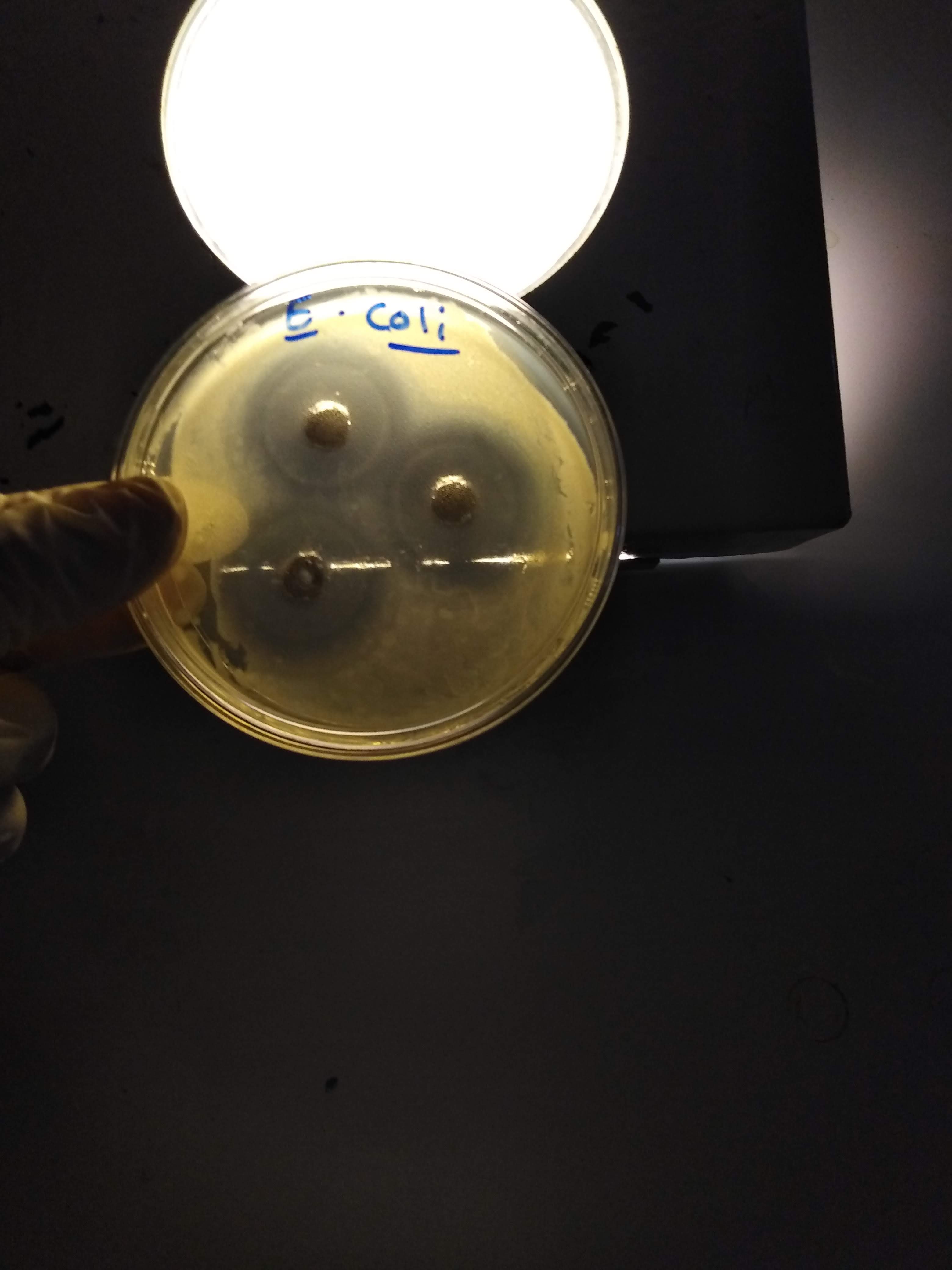

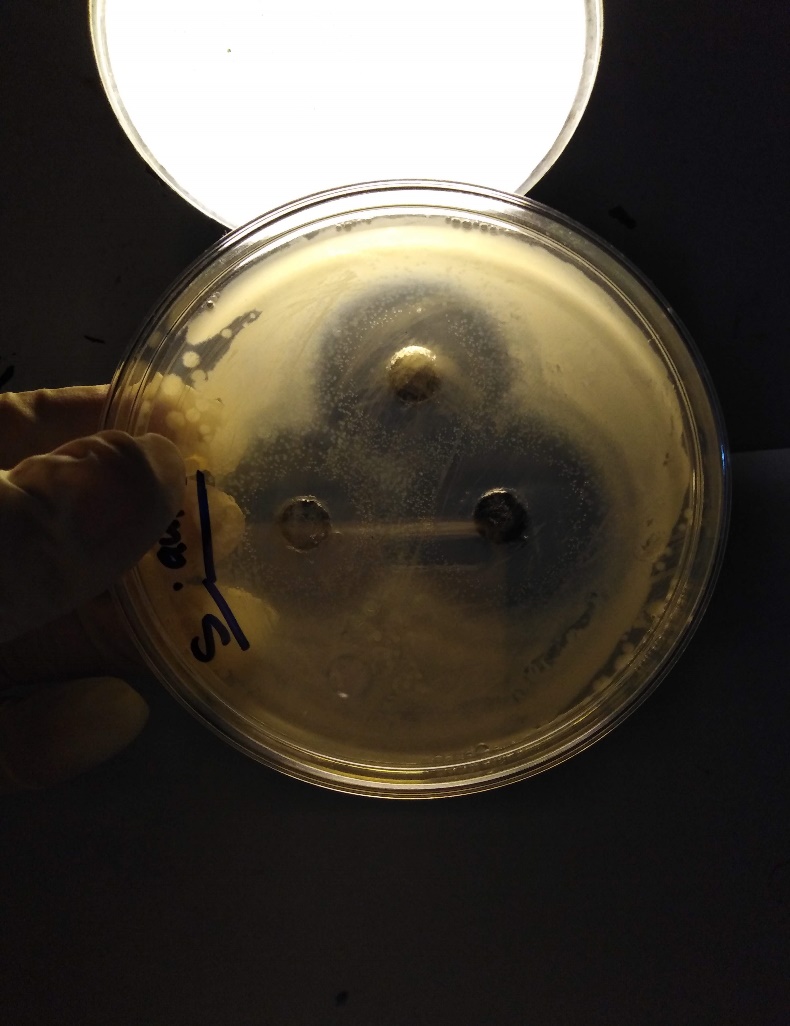


A2

B2

F

igure

**S2 Fig** Antibiotics sensitivity test and Antimicrobial activity of ZnO/CuO NPs after calcination at 550°C for 3 hrs against pathogenic bacteria in agar diffusion test; E. coli (A1,2) and S. aureus (B1,2)
