# Supplementary material for: Inactivating pathogenic bacteria in greywater by biosynthesized Cu/Zn nanoparticles from secondary metabolite of Aspergillus iizukae; optimization, mechanism and techno economic analysis
Source: PLoS One. 2019 Sep 12;14(9):e0221522. doi: 10.1371/journal.pone.0221522 (PMC6742378; doi:10.1371/journal.pone.0221522)
Supplement: S1 Table — (DOCX) [file pone.0221522.s003.docx]

**S1 Table** Coded and un-coded levels of the independent factors investigated in the present work

| **Factor** | **Symbol** | **Level** | | |
| --- | --- | --- | --- | --- |
|  |  | **Low (-1)** | **Middle (0)** | **High (+1)** |
| **ZnO/CuO NPs concentration (mg mL^-1^)** | $x_{1}$ | 0.01 | 0.055 | 0.1 |
| **Time (min)** | $x_{2}$ | 10 | 35 | 60 |
| **pH** | $x_{3}$ | 6 | 7 | 8 |
