# Supplementary material for: Inactivating pathogenic bacteria in greywater by biosynthesized Cu/Zn nanoparticles from secondary metabolite of Aspergillus iizukae; optimization, mechanism and techno economic analysis
Source: PLoS One. 2019 Sep 12;14(9):e0221522. doi: 10.1371/journal.pone.0221522 (PMC6742378; doi:10.1371/journal.pone.0221522)
Supplement: ‎S2 Table — (DOCX) [file pone.0221522.s004.docx]

**S2 Table** Central composite design arrangement and responses for inactivation of *E. coli* and *S. aureus* in greywater using bimetallic Zn/Cu NPs

| **Run** | $\boldsymbol{x}_{\boldsymbol{1}}$ | $\boldsymbol{x}_{\boldsymbol{2}}$ | $\boldsymbol{x}_{\boldsymbol{3}}$ | $\boldsymbol{y}_{\boldsymbol{1}}$ **(Log reduction)** | | $\boldsymbol{y}_{\boldsymbol{2}}$ **(log reduction)** |  |
| --- | --- | --- | --- | --- | --- | --- | --- |
|  |  |  |  | Observed | Predicted | Observed | Predicted |
| 1 | 0.000 | -1.682 | 0.000 | 3.5 | 3.65 | 2.35 | 2.79 |
| 2 | 0.000 | 0.000 | 0.000 | 5.87 | 5.44 | 4.28 | 4.71 |
| 3 | 0.000 | 0.000 | 0.000 | 5.52 | 5.44 | 5.21 | 4.71 |
| 4 | 0.000 | 1.682 | 0.000 | 5.6 | 5.74 | 5.3 | 5.27 |
| 5 | -1.682 | 0.000 | 0.000 | 1.24 | 1.76 | 1.004 | 1.83 |
| 6 | 0.000 | 0.000 | 0.000 | 5.39 | 5.44 | 4.96 | 4.71 |
| 7 | 0.000 | 0.000 | 1.682 | 4.95 | 5.32 | 3.23 | 3.99 |
| 8 | 1.682 | 0.000 | 0.000 | 5.9 | 5.67 | 5.89 | 5.48 |
| 9 | 0.000 | 0.000 | 0.000 | 5.69 | 5.44 | 5.04 | 4.71 |
| 10 | 1.000 | 1.000 | 1.000 | 6 | 5.95 | 4.4 | 4.69 |
| 11 | 0.000 | 0.000 | 0.000 | 4.99 | 5.44 | 4.46 | 4.71 |
| 12 | -1.000 | 1.000 | 1.000 | 4.22 | 3.88 | 3.89 | 3.04 |
| 13 | 1.000 | -1.000 | -1.000 | 4.8 | 4.93 | 3.56 | 4.12 |
| 14 | -1.000 | -1.000 | -1.000 | 2.5 | 2.34 | 2 | 1.42 |
| 15 | 0.000 | 0.000 | 0.000 | 5.22 | 5.44 | 4.37 | 4.71 |
| 16 | 1.000 | -1.000 | 1.000 | 5.69 | 5.66 | 5.78 | 5.25 |
| 17 | 1.000 | 1.000 | -1.000 | 6 | 6.16 | 5.98 | 6.12 |
| 18 | 0.000 | 0.000 | -1.682 | 5.32 | 5.25 | 4.98 | 4.63 |
| 19 | -1.000 | 1.000 | -1.000 | 4.7 | 4.53 | 4.7 | 4.93 |
| 20 | -1.000 | -1.000 | 1.000 | 3 | 2.63 | 2.52 | 2.09 |

$x_{1}$ (Zn/Cu NPs concentration (mg mL^-1^); $x_{2}$ (Time, min); $x_{3}$ (pH), $y_{1}$ (*E. coli*); $y_{2} (S. aureus )$
