# Supplementary material for: Inactivating pathogenic bacteria in greywater by biosynthesized Cu/Zn nanoparticles from secondary metabolite of Aspergillus iizukae; optimization, mechanism and techno economic analysis
Source: PLoS One. 2019 Sep 12;14(9):e0221522. doi: 10.1371/journal.pone.0221522 (PMC6742378; doi:10.1371/journal.pone.0221522)
Supplement: S3 Table — (DOCX) [file pone.0221522.s005.docx]

**S3 Table** Regression coefficient and their significance of the quadratic model for inactivating *E. coli* and *S. aureus* seeded in greywater using bimetallic (Zn/Cu) NPs

| **Factor** | **Coefficient** | | **Standard Error** | | **F value** | | **P value** | |
| --- | --- | --- | --- | --- | --- | --- | --- | --- |
|  | $\boldsymbol{y}_{\boldsymbol{1}}$ | $\boldsymbol{y}_{\boldsymbol{2}}$ | $\boldsymbol{y}_{\boldsymbol{1}}$ | $\boldsymbol{y}_{\boldsymbol{2}}$ | $\boldsymbol{y}_{\boldsymbol{1}}$ | $\boldsymbol{y}_{\boldsymbol{2}}$ | $\boldsymbol{y}_{\boldsymbol{1}}$ | $\boldsymbol{y}_{\boldsymbol{2}}$ |
| **Model** | 5.44 | 4.71 | 0.15 | 0.27 | 25.16 | 7.68 | < 0.0001 | 0.0019 |
| $\mathbf{X}_{\mathbf{1}}$ | 1.16 | 1.09 | 0.100 | 0.18 | 136.59 | 35.61 | < 0.0001 | 0.0001 |
| $\mathbf{X}_{\mathbf{2}}$ | 0.62 | 0.74 | 0.100 | 0.18 | 38.65 | 16.43 | < 0.0001 | 0.0023 |
| $\mathbf{X}_{\mathbf{3}}$ | 0.021 | -0.19 | 0.100 | 0.18 | 0.045 | 1.09 | 0.8368 | 0.3212 |
| $\mathbf{X}_{\mathbf{1}}\mathbf{X}_{\mathbf{2}}$ | -0.24 | -0.38 | 0.13 | 0.24 | 3.36 | 2.54 | 0.0966 | 0.1422 |
| $\mathbf{X}_{\mathbf{1}}\mathbf{X}_{\mathbf{3}}$ | 0.11 | 0.12 | 0.13 | 0.24 | 0.70 | 0.24 | 0.4231 | 0.6354 |
| $\mathbf{X}_{\mathbf{2}}\mathbf{X}_{\mathbf{3}}$ | -0.23 | -0.64 | 0.13 | 0.24 | 3.22 | 7.28 | 0.1029 | 0.0224 |
| $\mathbf{X}_{\mathbf{1}}$**^2^** | -0.61 | -0.37 | 0.097 | 0.18 | 39.42 | 4.42 | < 0.0001 | 0.0618 |
| $\mathbf{X}_{\mathbf{2}}$**^2^** | -0.26 | -0.24 | 0.097 | 0.18 | 7.33 | 1.82 | 0.0221 | 0.2072 |
| $\mathbf{X}_{\mathbf{3}}$**^2^** | -0.056 | -0.14 | 0.097 | 0.18 | 0.33 | 0.62 | 0.5780 | 0.4481 |

$x_{1}$ (Zn/Cu NPs concentration (mg mL-1); $x_{2}$ (Time, min); $x_{3}$ (pH), $y_{1}$ (E. coli); $y_{2} (S. aureus )$
