# Supplementary material for: Inactivating pathogenic bacteria in greywater by biosynthesized Cu/Zn nanoparticles from secondary metabolite of Aspergillus iizukae; optimization, mechanism and techno economic analysis
Source: PLoS One. 2019 Sep 12;14(9):e0221522. doi: 10.1371/journal.pone.0221522 (PMC6742378; doi:10.1371/journal.pone.0221522)
Supplement: ‎S4 Table — (DOCX) [file pone.0221522.s006.docx]

**S4** **Table** The best operating parameters for inactivation of *E. coli* and *S. aureus* in greywater using bimetallic Zn/Cu NPs

| $\boldsymbol{x}_{\boldsymbol{1}}$ | $\boldsymbol{x}_{\boldsymbol{2}}$ | $\boldsymbol{x}_{\boldsymbol{3}}$ | $\boldsymbol{y}_{\boldsymbol{1}}$ **(R%)** | | $\boldsymbol{y}_{\boldsymbol{2}}$ **(R%)** |  |
| --- | --- | --- | --- | --- | --- | --- |
|  |  |  | Observed | Predicted | Observed | Predicted |
| 0.028 | 60 min | 6 | 5.6 | 5.3 | 5.2 | 5.4 |

$y_{1} ($*E. coli*), $y_{2} ($*S. aureus*)
